# 1136. A Systematic Literature Review of Disparities That May Influence Health Equity in Invasive Meningococcal Disease Prevention in the US

**DOI:** 10.1093/ofid/ofad500.977

**Published:** 2023-11-27

**Authors:** Shahina Begum, Oscar Herrera-Restrepo, Catherine Rolland, Sneha Purushotham, Linda Hortobagyi, Zeki Kocaata

**Affiliations:** GSK, London, England, United Kingdom; GSK, London, England, United Kingdom; Evidera, London, England, United Kingdom; Evidera, London, England, United Kingdom; GSK, London, England, United Kingdom; GSK, London, England, United Kingdom

## Abstract

**Background:**

Meningococcal serogroup A, C, W, Y (MenACWY) and B (MenB) vaccines are recommended in the US to prevent invasive meningococcal disease (IMD), a rare but life-threatening disease. Yet the suboptimal uptake of and adherence to these vaccines may relate to inequities for healthcare access. This systematic literature review (SLR) synthesized the US evidence on disparities associated with IMD prevention.

**Methods:**

Embase, MEDLINE and six conferences were searched and handpicked studies included from 01/01/2012 to 23/08/2022. Studies were screened twice against inclusion criteria (Table 1) via a standardised form. Newcastle Ottawa Scale was used for quality assessment. Synthesis findings reported here were from a broader SLR on IMD risk, prevention and control.

**Figure:**
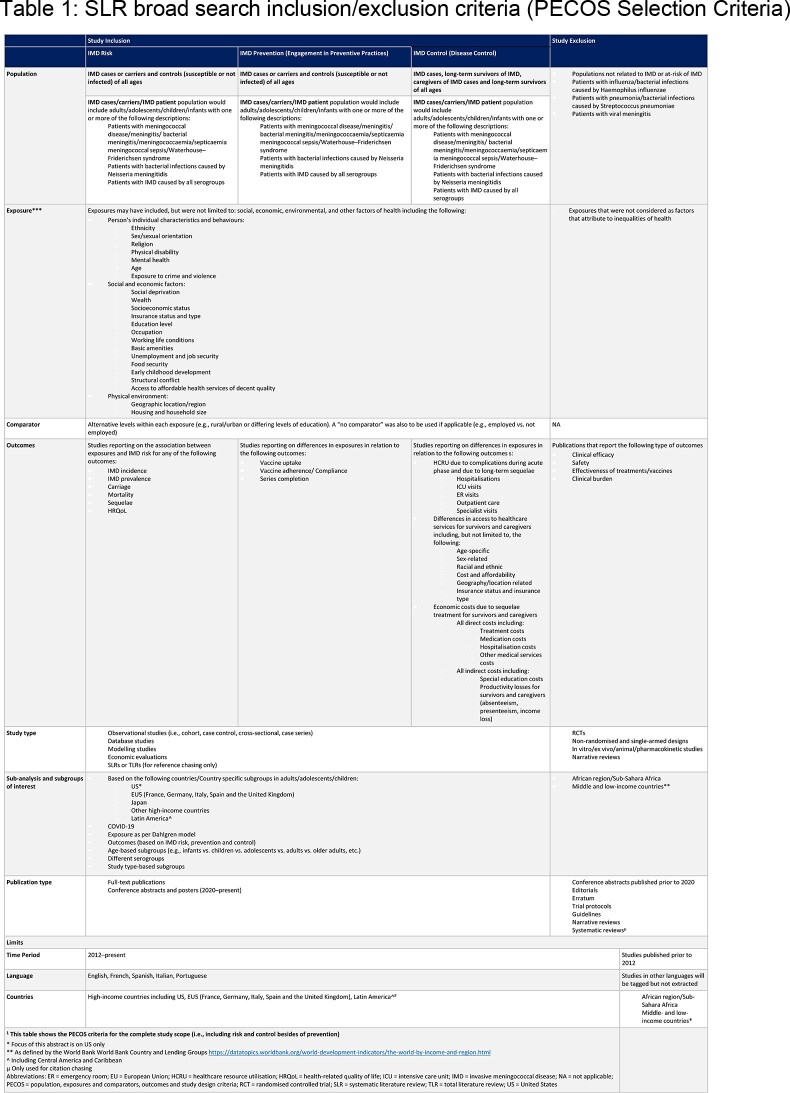

**Results:**

The SLR found 26 studies, 14 relevant for prevention (Table 2-3). MenACWY series completion had higher odds for adolescents with a family annual income >$75,000 vs. ≤$30,000. Individuals from families below poverty status (annual income ≤$75,000 in 2021) had lower MenB vaccination coverage rates. MenACWY booster compliance was lower in uninsured vs. insured adolescents. Higher odds for ≥1 dose of MenB vaccine were observed if individuals had Medicaid vs. private insurance. For parents/guardians of ≥1 dependent of 16-19 years old, with some insurance was significantly associated with MenB vaccine initiation vs. no insurance. Adolescents living outside a metropolitan statistical area had lower vaccination coverage with ≥1 MenACWY than adolescents in MSA principal cities. MenB series completion rates were lower in rural vs. urban areas in commercial insured populations. Non-Hispanic Black and Hispanic population were more likely to be vaccinated for MenB compared to non-Hispanic Whites, however one study found the opposite. MenB series completion were lower in the Black versus White Medicaid populations.

**Figure:**
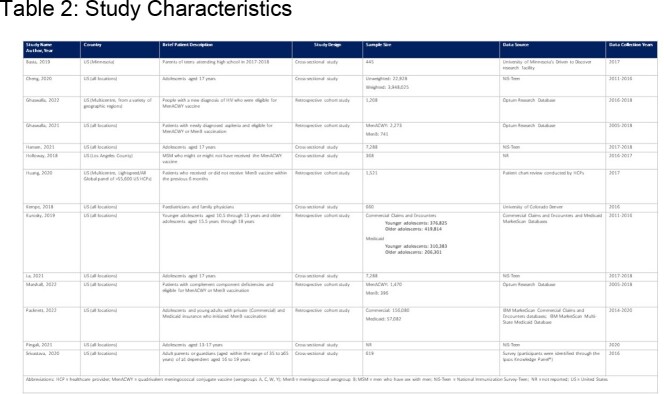

**Figure:**
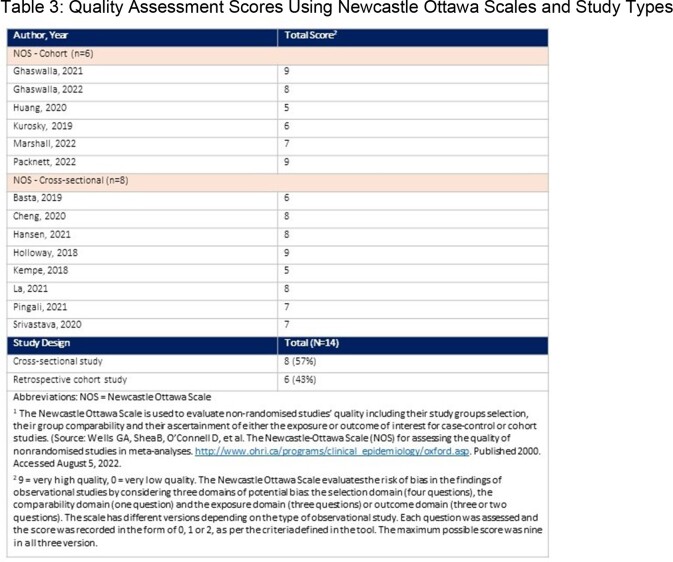

**Conclusion:**

Disparities in IMD prevention were reported for race/ethnicity, geographical factors, income/poverty level and health insurance status. Simplification of current recommendation (e.g., combination vaccine with increased convenience and reduced injections in the IMD vaccination schedule) could improve low uptake and adherence in disadvantaged groups.

**Disclosures:**

**Shahina Begum**, GSK: Employee **Oscar Herrera-Restrepo, PhD**, GSK: Stocks/Bonds **Catherine Rolland, PhD**, Evidera: Advisor/Consultant **Sneha Purushotham, MSc**, Evidera: Advisor/Consultant **Linda Hortobagyi, MSc**, GSK: Contractor **Zeki Kocaata, PhD**, GSK: Stocks/Bonds

